# Dominant collagen XII mutations cause a distal myopathy

**DOI:** 10.1002/acn3.50882

**Published:** 2019-09-11

**Authors:** Payam Mohassel, Teerin Liewluck, Ying Hu, Daniel Ezzo, Tracy Ogata, Dimah Saade, Sarah Neuhaus, Véronique Bolduc, Yaqun Zou, Sandra Donkervoort, Livija Medne, Charlotte J. Sumner, P. James B. Dyck, Klaas J. Wierenga, Gihan Tennekoon, Richard S. Finkel, Jiani Chen, Thomas L. Winder, Nathan P. Staff, A. Reghan Foley, Manuel Koch, Carsten G. Bönnemann

**Affiliations:** ^1^ National Institutes of Health, NINDS, NNDCS Bethesda Maryland; ^2^ Department of Neurology Mayo Clinic Rochester Minnesota; ^3^ Roberts Individualized Medical Genetics Center, Division of Human Genetics Children’s Hospital of Philadelphia Philadelphia Pennsylvania; ^4^ Departments of Neurology and Neuroscience Johns Hopkins University School of Medicine Baltimore Maryland; ^5^ Department of Clinical Genomics Mayo Clinic Jacksonville Florida; ^6^ Division of Neurology Children's Hospital of Philadelphia Philadelphia Pennsylvania; ^7^ Department of Pediatrics Nemours Children’ Health System Orlando Florida; ^8^ Division of Genomic Diagnostics Children’s Hospital of Philadelphia Philadelphia Pennsylvania; ^9^ Invitae Corporation San Francisco California; ^10^ Institute for Dental Research and Oral Musculoskeletal Biology, Center for Biochemistry, Medical Faculty University of Cologne Cologne Germany

## Abstract

**Objective:**

To characterize the natural history and clinical features of myopathies caused by mono‐allelic, dominantly acting pathogenic variants in *COL12A1.*

**Methods:**

Patients with dominant *COL12A1*‐related myopathies were characterized by history and clinical examination, muscle imaging, and genetic analysis. Pathogenicity of the variants was assessed by immunostaining patient‐derived dermal fibroblast cultures for collagen XII.

**Results:**

Four independent families with childhood‐onset weakness due to novel, dominantly acting pathogenic variants in *COL12A1* were identified. Adult patients exhibited distal‐predominant weakness. Three families carried dominantly acting glycine missense variants, and one family had a heterozygous, intragenic, in‐frame deletion of exon 52 of *COL12A1*. All pathogenic variants resulted in increased intracellular retention of collagen XII in patient‐derived fibroblasts as well as loss of extracellular, fibrillar collagen XII deposition. Since haploinsufficiency for *COL12A1* is largely clinically asymptomatic, we designed and evaluated small interfering RNAs (siRNAs) that specifically target the mutant allele containing the exon 52 deletion. Immunostaining of the patient fibroblasts treated with the siRNA showed a near complete correction of collagen XII staining patterns.

**Interpretation:**

This study characterizes a distal myopathy phenotype in adults with dominant *COL12A1* pathogenic variants, further defining the phenotypic spectrum and natural history of *COL12A1*‐related myopathies. This work also provides proof of concept of a precision medicine treatment approach by proposing and validating allele‐specific knockdown using siRNAs specifically designed to target a patient’s dominant *COL12A1* disease allele.

## Introduction

Collagen XII is a homotrimeric, FACIT (Fibril Associated Collagen with Interrupted Triple helices) extracellular matrix protein (reviewed here^1^). The collagen XII alpha chain encoded by *COL12A1* is expressed widely in bone, myotendinous junction, tendon, and other connective tissues during embryonic development. Postnatally, its expression becomes more restricted to dense connective tissues and is highly induced by tissue tensile stress and inflammation. Produced primarily by fibroblasts, collagen XII is typically co‐expressed with fibrillar collagens (e.g., collagen I, III, V) where it plays a role in cross‐linking and organizing collagen fibrils by interacting with other extracellular matrix (ECM) proteins (e.g., tenascin X).

Pathogenic variants in *COL12A1* cause a rare form of congenital connective tissue/myopathy overlap syndrome,^2^, ^3^ subsumed under the classification of “myopathic Ehlers‐Danlos syndrome (EDS).”^4^ Both recessive and dominant modes of inheritance have been described in patients with *COL12A1‐*related myopathic EDS. In general, recessively inherited, bi‐allelic loss‐of‐function variants cause a severe congenital disease characterized by hypotonia, global muscle weakness and atrophy, respiratory insufficiency, and striking joint hyperlaxity concurrent with contractures in some of the larger joints, precluding independent ambulation. In contrast, haploinsufficiency typically does not manifest with a detectable clinical phenotype in children or adults except possibly for mild joint hyperlaxity. Heterozygous variants with a dominant‐negative pathogenic effect on collagen XII fibrillar assembly have also been described and generally cause a much milder phenotype compared to the bi‐allelic loss of function situation, characterized by mild motor developmental delays, hypotonia, mild proximal weakness, and joint hyperlaxity.^2^, ^3^, ^5^, ^6^ Similar to other collagens, disruption of the triple helical (TH), Gly‐X‐Y containing domain structure is characteristic of dominantly acting pathogenic variants (typically in‐frame exon skipping or missense glycine substitutions). To date, only few patients, mostly children, have been reported in the literature with dominantly acting *COL12A1* pathogenic variants, and the full phenotypic spectrum of this disease is not yet delineated.

Here, we report four families with novel, dominantly acting, heterozygous pathogenic variants in *COL12A1* resulting in a connective tissue/myopathy overlap syndrome. In particular, we report a distal predominant pattern of weakness in three adult patients from two independent families, thus expanding and specifying the phenotypic spectrum of the disease. While three families carried dominantly acting glycine missense variants, in one family we found an intragenic, in‐frame deletion of exon 52 of *COL12A1*. For this variant, we show that allele‐specific knockdown using short interfering RNA (siRNA) designed to selectively target the pathogenic allele is a feasible therapeutic strategy, which restores fibril‐associated collagen XII distribution in the ECM in patient‐derived dermal fibroblast cultures. Thus, we highlight the importance of precision in molecular diagnosis of patients and the feasibility of rapid translation of suitable pathogenic allele‐directed therapeutic strategies, especially for conditions caused by gain‐of‐function or dominant‐negative‐acting variants amenable to allele‐specific knockdown.

## Material and Methods

### Patient recruitment and sample collection

All patients presented with a history of early‐onset myopathy and underwent detailed clinical examination. DNA and tissues (e.g., muscle, skin) and medical records were obtained based on standard procedures. For research studies, written informed consent and age appropriate assent was obtained from all participants. Ethical approval was obtained from the NIH, National Institute of Neurological Disorders and Stroke (NINDS), Institutional Review Broad (Protocol 12‐N‐0095).

### Imaging studies

Conventional T1‐weighted spin echo and short tau inversion recovery (STIR) of the lower extremities on a 3.0T Achieva Phillips MRI system were obtained. Muscle ultrasound images were obtained using a Siemens S2000 with a 15MHz linear probe and rated based on the Heckmatt scale.^7^

### Exome sequencing and analysis

Exome sequencing was performed on genomic DNA extracted from blood. Quartet exome sequencing in family 1 was performed through the NIH Intramural Sequencing Center (NISC) using the Illumina (San Diego, CA) TruSeq Exome Enrichment Kit and Illumina HiSeq 2500 sequencing instruments. Variants were analyzed using *Seqr* (Center for Mendelian Genomics) and searched for in dbSNP, NHLBI EVS, Exome Aggregation Consortium (ExAC Browser and GEM.app).^8^

For patient 2, all coding exons (exons 2–66) and the 10–20 bases flanking intronic sequences of *COL12A1* (NM_004370.5) were captured with oligonucleotide baits (Agilent Technologies, Santa Clara, CA; Roche, Pleasanton, CA; IDT, Coralville, IA). Next‐generation sequencing (NGS) was performed on Illumina platforms (San Diego, CA), and quality control standards were implemented to achieve a minimum of 50× and an average of 350× depth‐of‐sequence read coverage. Sequence and copy number variant (CNV) analyses were performed with validated NGS methods, allowing for concurrent analysis of sequence variants and exonic CNVs. Diagnostic findings were confirmed by alternate methods.^9^ Patient 3 and 4 underwent diagnostic clinical whole‐exome sequencing via a CLIA‐certified lab (GeneDx).

### Fibroblast cultures, RNA extraction, RT‐PCR, and qPCR

Fibroblasts derived from skin biopsy of patients and controls were grown in high‐glucose Dulbecco’s modified Eagle’s medium (DMEM, Sigma, Poole, UK) supplemented with 10% FBS and penicillin/streptomycin. Using, patient derived skin fibroblast cultures as starting material, Qiagen miRNeasy kit was used for RNA isolation following the manufacturer’s protocol. QIAzol (Qiagen) was used for initial RNA isolation. The aqueous phase was isolated after addition of chloroform and the RNA was dissolved in ethanol and cleaned on spin columns and eluted in RNase free water. Qscript XLT cDNA SuperMix (Quanta bio) was used to produce cDNA from the isolated RNA following the manufacturer’s instructions. Endpoint PCR followed by sequencing was performed the cDNA.^3^ For allele‐specific knockdown experiments, endpoint PCR was performed using Advantage 2 (Takara Bio) using cDNA, E51F and E53R primers, flanking the deleted exon 52. PCR products were electrophoresed on a 2% agarose gel in TAE buffer.

Real‐Time PCR (qPCR) was performed using FastStart Universal Master‐mix (Roche) and wild‐type and mutant del52 *COL12A1* allele‐specific primers (probe #47 ‐‐Universal probe library, Roche). qPCR was run in triplicates. Linear fold change values were calculated using the ΔΔcycle threshold (ΔΔCt) method with *PGK1* (probe #67) as an internal control, averaged from the triplicates, and normalized to untreated cells from each cell line. All primer sequences can be found in Table S1.

### gDNA PCR and sequencing

Overlapping gDNA primers were designed to specifically screen for large genomic deletions leading to apparent exon 52 deletion in *COL12A1* in Family 1. Endpoint PCR was performed using KAPA‐HiFi ready mix (KAPA Biosystems) per manufacturer’s specifications. Primer sequences can be found in Table S1.

### siRNA transfection

RNAiMax (Invitrogen) was used as a liposomal transfection agent in antibiotic‐free media and manufacturer recommendations were followed. Two siRNAs were designed and purchased (Dharmacon Inc.) to test targeting the mutant allele in Family 1. A nontargeting siRNA (Dharmacon Inc., D‐001810‐01) was used as a negative control (Fig. S1).

### Immunostaining and microscopy

Fibroblasts from patient and controls were grown to confluence in glass chamber slides over 5 days, supplemented with vitamin C (50 *µ*g/mL) from day 3–5. For transfection experiments, the cells were transfected on the day of plating (day 0) and re‐transfected on day 3. After 5 days, cells were washed in PBS, and fixed using 4% PFA at room temperature for 15 min. After washing in PBS, they were blocked in 10% FBS in PBS with Triton‐X (0.1% or 0.5%) for 1 h at room temperature. Primary antibody (anti‐Collagen XII raised in Guinea pig at 1:1000– Manuel Koch, Cologne, Germany, and rabbit anti‐fibronectin, Sigma Aldrich at 1:800) incubation was performed overnight at 4°C. Secondary antibodies (Alexa 488 anti‐rabbit and Alexa 568 anti‐Guinea pig) were incubated for 1 h at 1:500 dilution at room temperature. After washing with PBS × 3 and staining with DAPI, the slides were coverslipped and viewed using a Leica TCS SP5 II confocal microscope.

## Results

### Clinical presentation

All patients (*n* = 6) with heterozygous *COL12A1* pathogenic variants reported mild muscle weakness without prominent clinical progression. Hyperlaxity in the small and large joints was noted in the majority of patients (5/6), including in two adults (Family 1, P3, P4). The pattern of weakness was variable and in pediatric patients (*n* = 3) manifested with both proximal and distal weakness. In adults (*n* = 3), clinically detectable weakness was generally limited to distal muscles. In the lower extremities, the anterior leg compartment was more affected than the posterior compartment. In the upper extremities, finger extensors and intrinsic hand muscles were selectively affected. Even in those patients who first sought an evaluation in adulthood (patients 1B, 1C, 2), childhood or congenital symptoms (e.g., hypotonia or mild motor developmental delays) were invariably recalled upon questioning, albeit without a clear distribution pattern. These reported symptoms appear to have improved, as most patients reported little to no limitations in motor activities in young adulthood (2nd and 3rd decade of life) but then developed mild symptomatic weakness beginning in the 4th decade of life or later. In addition to muscle weakness, mild ankle contractures were noted in one patient (P1A), and the majority of patients had pes planus deformities in the feet. No cardiac or pulmonary manifestations were detected. The inheritance in family 1 was consistent with an autosomal dominant pattern. Family members of P2 were not clinically evaluated. P3 and P4 had *de novo* pathogenic variants and reported noncontributory family histories. The details of the clinical presentation, signs and symptoms, and relevant laboratory findings are summarized in Table 1 and Table S2.

**Table 1:** General phenotype of patients with heterozygous, dominantly acting, *COL12A1* pathogenic variants.

Patient	1A^a^	1B	1C	2	3	4
Sex	M	F	M	M	M	M
Recognized Onset	Congenital	Congenital	Congenital	4 years	Congenital	Congenital
Age	5	37	33	62	4	3
Motor Development	Mild gross motor delay	Mild gross motor delay	Mild gross motor delay	Normal	Mild gross and fine motor delay	Mild gross and fine motor delay
Pattern of Weakness (MRC)	Neck flexion (2/5), proximal and distal weakness (4/5)	Distal (4+/5)	Distal (4/5)	Distal (4/5)	Mild distal and proximal weakness	Head lag, mild proximal and distal weakness
Joint Hyperlaxity	Distal and proximal	Distal and proximal	Distal and proximal	None	Distal and proximal	Distal and proximal
FVC	95%	104%	96%	115%	81%	ND
CK (U/L)	174	60	101	106	103	99
Mutation	c.7951‐630_8100+991 del1771ins10			c.8276G>A, p.Gly2759Asp	c.8453G>A, p.Gly2818Glu	c.8065 G>A, p.Gly2689Arg

a 1A is 1B’s son, and 1C is 1B’s brother. FVC, forced vital capacity (reported as percent predicted); ND, not done.

### Phenotypic analyses

Electrophysiologic studies, when available, showed mild reductions in CMAP amplitude in distal muscles. EMG showed myopathic motor unit action potentials (MUAPs) in most patients. In addition, a few isolated neurogenic appearing features (e.g., large amplitude MUAPs or abnormal spontaneous activity) were noted sporadically in a few patients. One patient (P2) had nonsustained myotonic discharges in distal muscles and as a result underwent genetic testing for myotonic dystrophy type 1 and 2, both of which were negative (Table S2).

**Figure 1:**
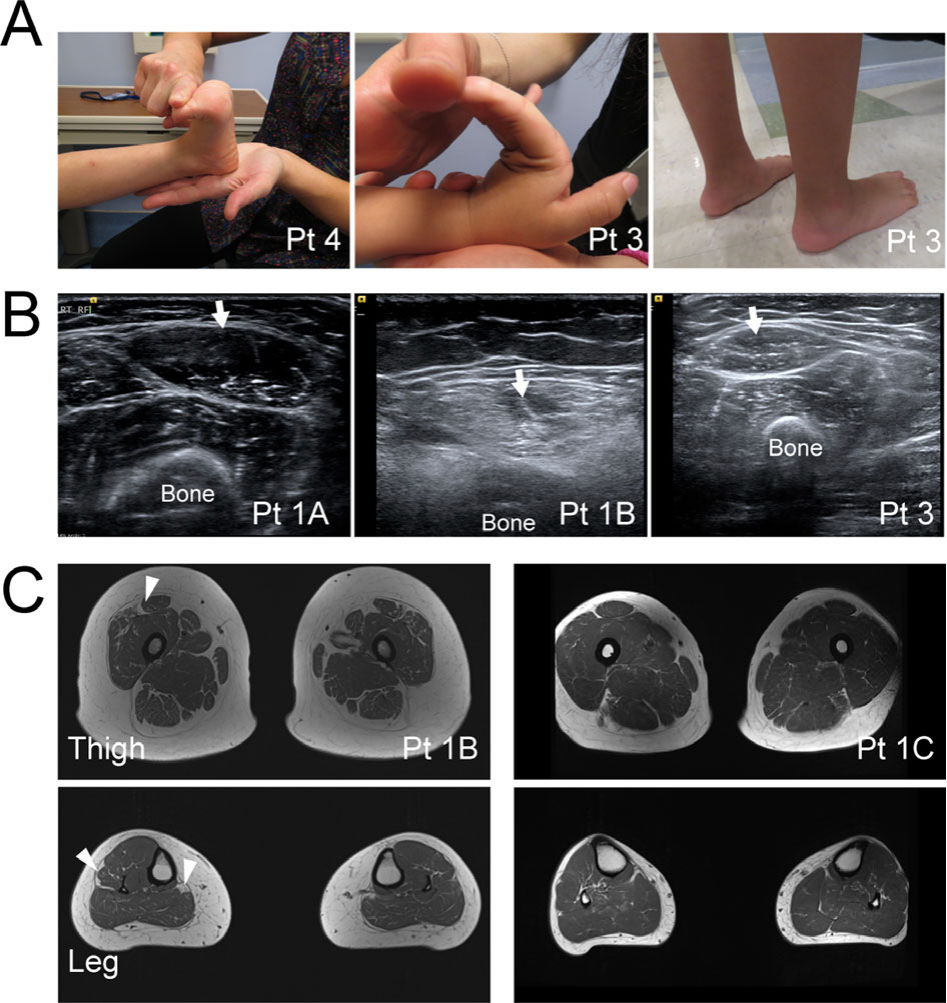
Clinical photos and muscle imaging. (A) Joint hyperlaxity (left and middle) and prominent calcanei (right) are common findings in patients with connective tissue disease, including dominant collagen XII‐related myopathic EDS. (B) Muscle Ultrasound shows normal appearing muscle in younger patients (1A, 3) and more pronounced increase in granular echogenicity in the rectus femoris muscle (arrows) in the older patient (1B). Notably, the central increase in echogenicity, characteristic of collagen VI‐related disorders is absent (1B). (C) Muscle MRI of the thigh and lower legs show mild increase in T1 weighted signal. This is most notable near the epimysium of rectus femoris, peroneus longus, and medial gastrocnemius muscles (arrowheads).

Muscle ultrasound generally showed a pattern of mild to moderately increased echogenicity with a concurrent granular and streak‐like appearance (Fig. 1B). Notably, the muscle fibers surrounding the central fascia of the rectus femoris muscle did not show a selective increase in echogenicity (referred to as a “central cloud”), which is typically seen in collagen VI‐related muscular dystrophies.^10^ Muscle MRI was obtained on two adult patients (P1B and 1C), demonstrating normal or only minimal changes in T1 signal in lower extremity muscles. Muscle atrophy and a distinctive appearance of increased epimysial fat was noted in select thigh (e.g., rectus femoris) and lower leg (e.g., peroneus longus) muscles (Fig. 1C).

**Figure 2:**
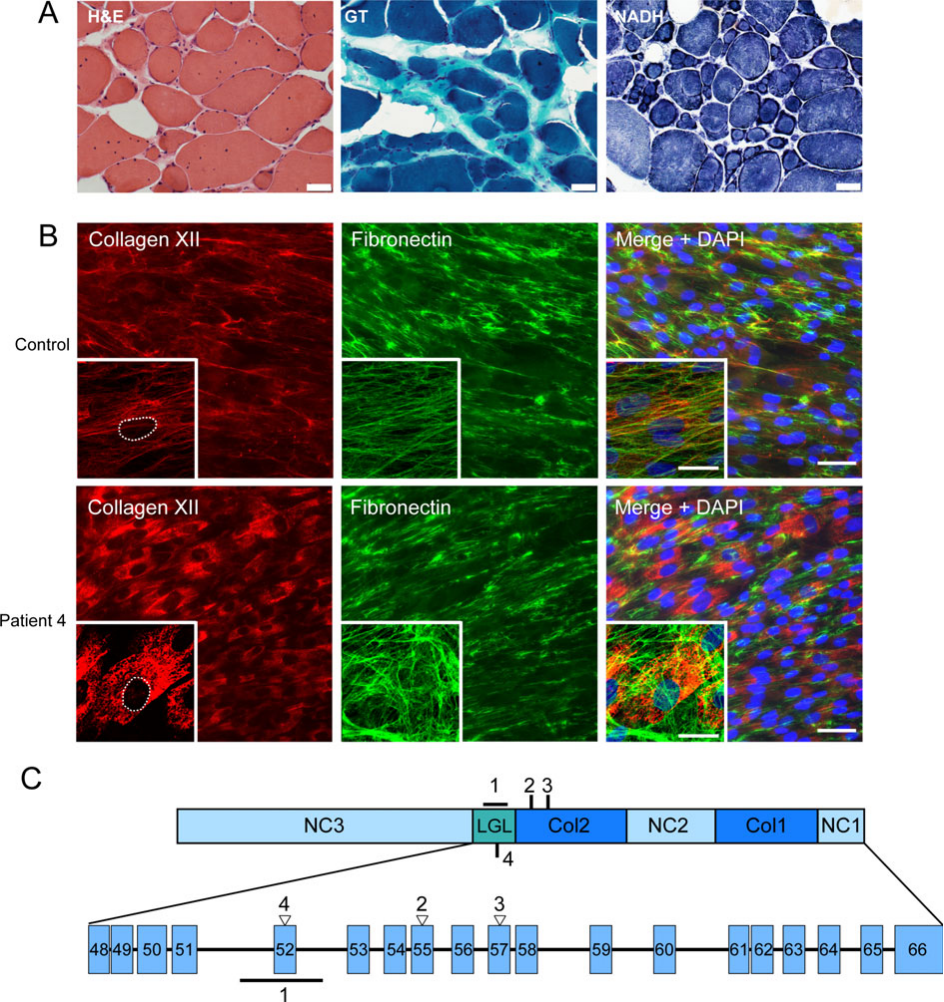
Muscle histology and dermal fibroblast immunostaining. (A) Muscle biopsy of patient 2 (tibialis anterior) shows increased internal nuclei, fiber size variability, and type 1 fiber predominance with numerous ring fibers. (B) Immunofluorescent images of skin dermal fibroblast cultures obtained from patient 4 compared to normal control. There is a near complete absence of extracellular fibrillar collagen XII (red) staining with prominent intracellular retention of collagen XII when compared against other extracellular matrix proteins, for example, fibronectin (green), scale bar 50 *µ*m. The insets show higher magnification, confocal microscope images of a single cell with the nucleus outlined, highlighting the significant intracellular retention of collagen XII in the patient cells; scale bar for insets 25 *µ*m (C) Schematic diagram of collagen XII single chain, its different domains, and *COL12A1* exons corresponding to the region to which the pathogenic variants from family 1–4 map. Empty triangle denotes glycine substitution. Line denotes deletion in family 1 with breakpoints in intron 51 and 52. Col = collagenous domain, NC = noncollagenous domain, LGL = laminin‐G like domain.

Muscle biopsy was obtained in two patients (P1B and P2). P1B muscle biopsy obtained from a clinically unaffected (by exam and imaging) proximal muscle and was reported to have a normal histologic appearance. P2 muscle biopsy showed an increase in internalized nuclei, myofiber size variability with type 1 fiber predominance and multiple ring fibers, (Fig. 2A). No neurogenic changes were appreciated.

### Molecular genetics and functional studies

All patients underwent CLIA‐certified next‐generation sequencing panel testing and/or research‐based whole‐exome sequencing. Except for variants in *COL12A1,* no disease‐causing variants in other known myopathy or connective tissue disease genes were detected. In P2, P3 and P4, panel testing or whole‐exome sequencing identified heterozygous *COL12A1* missense variants, none of which are reported in unaffected individuals in the ExAC database (Table 1).

Skin‐derived fibroblasts express collagen XII, which can be visualized using immunofluorescent microscopy. To assess the functional consequence of the identified variants, patients also underwent a diagnostic skin biopsy. In contrast to the controls, all cell lines from the patients had near complete absence of extracellular fibrillar staining and near exclusive intracellular staining for collagen XII (Fig. 2B and Fig. S2). This abnormal pattern of staining has been previously reported in pathogenic, dominantly acting collagen XII mutations^2^, ^3^ and provides cell‐based, functional confirmation of pathogenicity of these novel mutations. In particular, these mutations all resulted in substitution of highly conserved glycine residues in the triple helical domain of collagen XII (P2,3) or in the laminin G‐like domain in the adjacent N‐terminal region (P4) (Fig. 2C).

**Figure 3:**
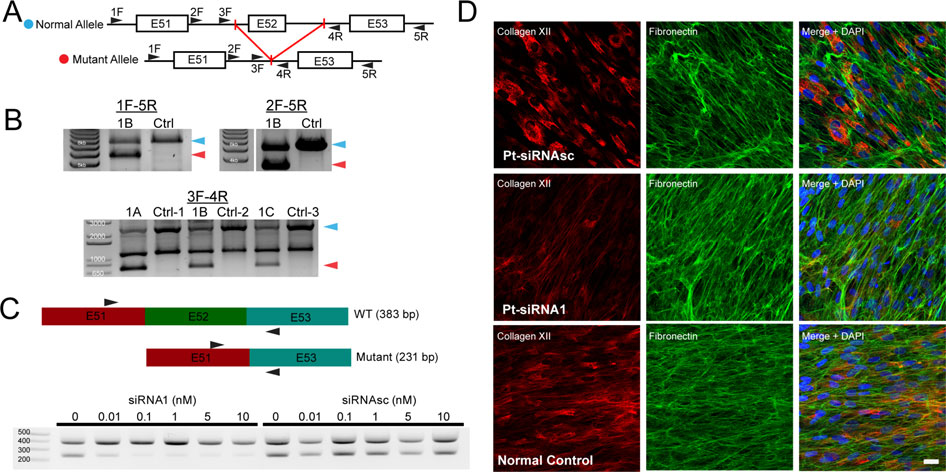
Identification of a large genomic deletion mutation in family 1 and siRNA mediated allele specific knockdown. (A) Schematic of the long‐range PCR approach to identify the suspected genomic deletion mutation in this region. (B) A ~ 1.8 kb heterozygous deletion between primers 1F and 5R is identified and further characterized between 3F and 4R primers. This heterozygous deletion encompassing exon 52 segregates with the disease phenotype in family 1. Ctrl‐1 = unaffected sibling of 1A, Ctrl‐2 = unaffected parent of 1A, Ctrl‐3 = normal control. (C) Schematic of the wild‐type and mutant cDNA in family 1 (top) and location of cDNA primers. Endpoint PCR of the cDNA from dermal fibroblasts before and after treatment with allele‐specific siRNA1 designed to target the exon 51–53 junction shows a dose dependent decrease in the pathogenic deleted allele. Control scrambled siRNAsc does not have an effect on either allele. (D) Immunofluorescent staining of P1b dermal fibroblasts after treatment with siRNA1 (0.1 nmol/L) but not control siRNAsc results in a prominent reduction of intracellular retention and restoration of the fibrillar pattern of extracellular collagen XII staining (red), similar to the normal control.

In family 1, whole‐exome sequencing results failed to detect any disease associated variants in *COL12A1*. Because of a strong clinical suspicion for collagen XII‐related myopathy or other similar connective tissue disorder, we also obtained a skin biopsy from P1B and stained the fibroblasts for collagen XII, which demonstrated a complete absence of extracellular fibrillar collagen XII staining and a near complete intracellular staining for collagen XII. Sequencing of collagen XII cDNA derived from skin fibroblasts identified a heterozygous deletion of exon 52, which is in‐frame of the coding sequence. Sequencing of the genomic DNA (gDNA) flanking this exon did not reveal any splice site or deep intronic mutations; however, sequencing of PCR products of the genomic region never showed heterozygosity for any known SNPs, even in the highly variable intronic regions. This observation suggested the presence of hemizygosity due to a large, heterozygous deletion within the genomic DNA, including exon 52. Thus, we designed primers encompassing this region in order to identify the breakpoints. Gel electrophoresis and sequencing of long‐range PCR products of the gDNA region flanking exon 52 then confirmed the presence of a heterozygous deletion (1771 bp) with breakpoints in intron 51 and 52 and insertion of a 10‐bp sequence (Fig. 3A and B). This mutation was not reported in the genome aggregation database (GnomAD) and segregated with the phenotype consistent with autosomal dominant inheritance pattern in this family (Fig. 3B, lower gel image).

### Treatment strategy

**Figure 4:**
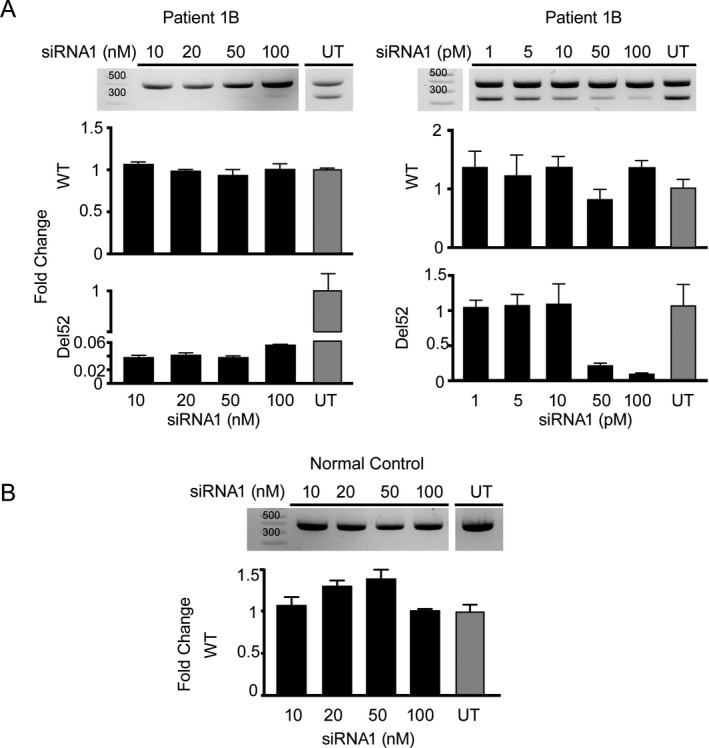
Evaluation of the effective dose range of siRNA1. (A) Left: Increasing siRNA1 dose to 100 nmol/L does not affect the normal allele as visualized on end point PCR (top) or real‐time PCR using wild‐type (WT) or mutant (del52) specific primers. Right: Decreasing the siRNA1 dose to pmol/L range confirms that maximal effects of allele specific knockdown are apparently achieved between 50 and 100 pmol/L. (**B)** High concentrations of the siRNA1 up to 100 nmol/L also do not affect the normal (WT) allele in normal control cells. Real‐time PCR for each siRNA1 concentration was performed in triplicates and the fold change values were averaged and normalized to the untreated samples. Bar height represents the mean fold change and error bar represents SEM (three technical replicates) for each condition.

Since haploinsufficiency of collagen XII does not cause a clinically significant phenotype in childhood or adulthood (as can be assessed in the heterozygous carriers of recessively acting null mutation),^3^ we explored allele‐specific knockdown using siRNA as a plausible approach to correct the pathogenic consequences of this deletion in order to re‐establish normal formation of fibrillar collagen XII in the  ECM. Using established criteria for designing effective siRNAs (reviewed here^11^), we designed and evaluated two siRNAs against the exon 51–53 junction to selectively target the mutant mRNA with exon 52 deletion in family 1 (Fig. S1). Indeed, transfection of patient skin‐derived fibroblasts with deletion‐specific siRNA efficiently reduced the quantity of the mutant del52 mRNA in a dose‐dependent manner (Fig. 3C) and restored the fibrillar pattern of collagen XII staining in the ECM deposited by the fibroblasts (Fig. 3D). Maximal effects were noted at 0.1 nmol/L concentration of one of the siRNAs in vitro; however, concentrations up to 100 nmol/L still did not affect the wild‐type allele mRNA levels in normal control or patient cells (Fig. 4), providing a large therapeutic window.

## Discussion

Here we have identified four novel, dominantly acting pathogenic variants in *COL12A1* in four unrelated families who clinically manifest a consistent phenotype of early‐onset hypotonia, wide‐spread joint hyperlaxity, and mild myopathy in childhood, with reported clinical improvement of muscle strength over time and recurrence of distal‐predominant weakness in adulthood, consistent with the recently established designation of myopathic EDS. It is notable that the pattern of weakness in adulthood seems to evolve to affect distal muscles more than proximal ones. When available, affected distal muscle needle EMG was mostly consistent with myopathic changes; however, small hand and foot muscles were rarely sampled and possibility of mild neurogenic appearing changes in them was not fully evaluated. Upon careful review of the literature, we found another multi‐generational family with dominantly inherited *COL12A1‐*related myopathy in which one older adult patient (aged 79) was described to have distal greater than proximal weakness.^6^ Thus, together with our findings, we propose dominantly inherited collagen XII myopathic EDS as part of the differential diagnosis of distal myopathy in adults. In addition, given the overall mild myopathic features, we surmise that some patients classified as hypermobile EDS might have *COL12A1* pathogenic variants in whom the myopathy has gone unnoticed, as recently reported.^12^

Even though phenotypic overlap between patients with *COL12A1*‐related myopathy and patients with Bethlem myopathy, a form of COL6‐related dystrophy due to mutations in collagen VI genes (*COL6A1, COL6A2,* or *COL6A3*) has been noted, several features help distinguish these disorders. Progressive muscle weakness with respiratory involvement, especially in older individuals, is more conspicuous in COL6‐related dystrophies, including the milder Bethlem myopathy;^13^, ^14^ however, distal predominant weakness is rarely reported for the COL6‐related disorders. From a clinical diagnostic standpoint, muscle imaging may be helpful in differentiating these disorders. Muscle ultrasound and MRI in the dominant collagen XII associated disease fail to illustrate the typical findings commonly seen in COL6‐related dystrophies (i.e., central cloud in rectus femoris muscle or “outside‐in” appearance of diseased muscle in vastus lateralis muscle) and can aid in differentiating these disorders in context with other phenotypic data. In addition, unlike COL6‐related dystrophies, muscle biopsy findings in dominant collagen XII‐related myopathy tend to be normal or have limited, mild myopathic features.^2^, ^3^ The preponderance of so‐called ring fibers in one of our patient muscle biopsies (Fig. 2A) is a peculiar finding. Ring fibers are described in myotonic dystrophy type 1 but also in animal models of tenotomy^15^ in which context they are postulated to arise due to loss of tensile forces on myofibers. We speculate that absence of fibril‐associated collagen XII from the tendon or myotendinous junction results in a situation akin to a chronic, mild, functional tenotomy and may explain this peculiar histologic finding. The muscle weakness resulting from *COL12A1* mutations could also to be due to such functional unloading of muscle.

While the majority of *COL12A1* mutations, especially glycine substitutions in the TH domain, are efficiently identified using next‐generation or whole‐exome sequencing, large deletions, duplications, inversions and deep intronic splice modulating variants can easily be missed. Illustrating this point, one multi‐generational family in our cohort was found to have a disease‐causing large (~1.8 kb) intragenic deletion, which was missed on diagnostic whole‐exome sequencing. The addition of high‐resolution microarray of genomic DNA, and RNA‐Seq analysis on patient‐derived fibroblasts or muscle biopsy to diagnostic platforms can assist in identification of these elusive variants, especially when functional assays such as immunostaining of fibroblasts can subsequently be used to assess and validate their pathogenicity. Clinical suspicion and recognition of the distinct disease phenotype continues to play a central role in interpretation of genetic tests and may prompt alternative approaches to molecular genetic analysis.^16^

Similar to other collagens, the glycine residues of the Gly‐X‐Y containing TH domain of collagen XII are evolutionarily highly conserved. Their substitutions with bulkier amino‐acids generally interferes with TH domain folding and thus overall collagen structure. Since triple helix formation proceeds from the C‐terminus to the N‐terminus in most collagens including collagen XII, the location of these mutations may determine their overall effects. Two of the disease‐causing, dominant glycine substitution variants in our patients are located in the Col2 domain, the more N‐terminal of the two interrupted TH domains in collagen XII (Fig. 2C). We hypothesize that the normal C‐terminal Col1 domain of the mutant chain likely initiates and is efficiently incorporated into the triple helix. However, the bulky missense variant in the N‐terminal Col2 domain then de‐stabilizes the three‐dimensional structure of the homotrimer or disrupts its protein‐protein interactions with fibrillar collagen associated proteins, for example, COMP^17^ or Tenascin‐X.^18^ Thus, glycine changing missense variants in this domain are predicted to exert a dominant‐negative effect, with 7/8 homotrimers predicted to contain at least one mutant chain.

The dominantly acting disease variant in family 1 results in an in‐frame internal deletion of exon 52, which is not part of the triple helical domain and encodes part of a laminin‐G like domain in the large NC3 domain (Fig. 2C). The glycine substitution in P4 (p. G2689R) is also in this exon. This region contains several cysteine residues and is hypothesized to be responsible for disulfide bridging of the single collagen XII chains and the overall stability of the homotrimer. Thus, deletion of exon 52 or glycine substitutions may disrupt the overall 3D structure of the homotrimer, its stability, and protein‐protein interactions. In addition, these changes may result in exposure of unpaired cysteines in the remaining chains within the homotrimer. In assembly of collagen type I,  unpaired cysteines have been suggested to result in misfolded proteins, which in turn may be trapped in the ER and trigger ER stress.^19^ The clear appearance of intracellular retention of collagen XII in patient fibroblasts with these mutations are consistent with this scenario. A similar mechanism has been proposed for another collagen XII mutation (p. R1965C) resulting in an unpaired cysteine in the NC3 domain.^2^ However, additional molecular studies are necessary to fully characterize the exact consequences of these mutation in the laminin G‐like domain on the collagen XII homotrimer with a focus on both structural changes in collagen XII superstructure and its cellular physiologic consequences.

Even though the exact molecular and pathophysiological consequences of dominantly acting pathogenic variants in *COL12A1* remain to be fully elucidated, gene or transcript directed therapeutic approaches can still be legitimately pursued as they are “agnostic” to downstream pathophysiological events. Following this rationale, we were able to successfully and selectively silence the exon 52 deletion disease allele using siRNA transfection. The siRNA was highly potent and maximally effective at 0.1 nmol/L concentration and restored the fibrillar association of collagen XII as assessed by immunofluorescent microscopy *in vitro.* Given the recent FDA approval of siRNAs for treatment of hereditary transthyretin amyloidosis^20^ and increasing experience with the safety of these and modified siRNAs as a class, we hope that such mutation‐specific therapies will have the potential for clinical translation and can be used as highly precise treatments for patients in clinical applications.

## Conflict of Interest

The authors do not have any relevant financial conflicts of interest.

## Supporting information


**Figure S1.** Schematic of *COL12A1* exons 51–53 and sequence of two siRNAs designed to specifically target exon 51‐53 junction, the mutant mRNA product in family 1.

**Figure S2.** Immunofluorescent images of skin dermal fibroblast cultures obtained from patient 1b, 2, 3, and 4 compared to two normal controls. There is a near complete absence of extracellular fibrillar collagen XII (red) staining with prominent intracellular retention of collagen XII when compared against other extracellular matrix proteins, for example, fibronectin (green).

**Table S1.** List of primers used for endpoint PCR and real‐time PCR (qPCR).

**Table S2.** Detailed description of clinical presentation, signs and symptoms, and relevant laboratory findings of patients with heterozygous, dominant, *COL12A1* pathogenic variants.
