# Supplementary figures and images for: Dominant collagen XII mutations cause a distal myopathy
Source: Ann Clin Transl Neurol. 2019 Sep 11;6(10):1980–8. doi: 10.1002/acn3.50882 (PMC6801183; doi:10.1002/acn3.50882)

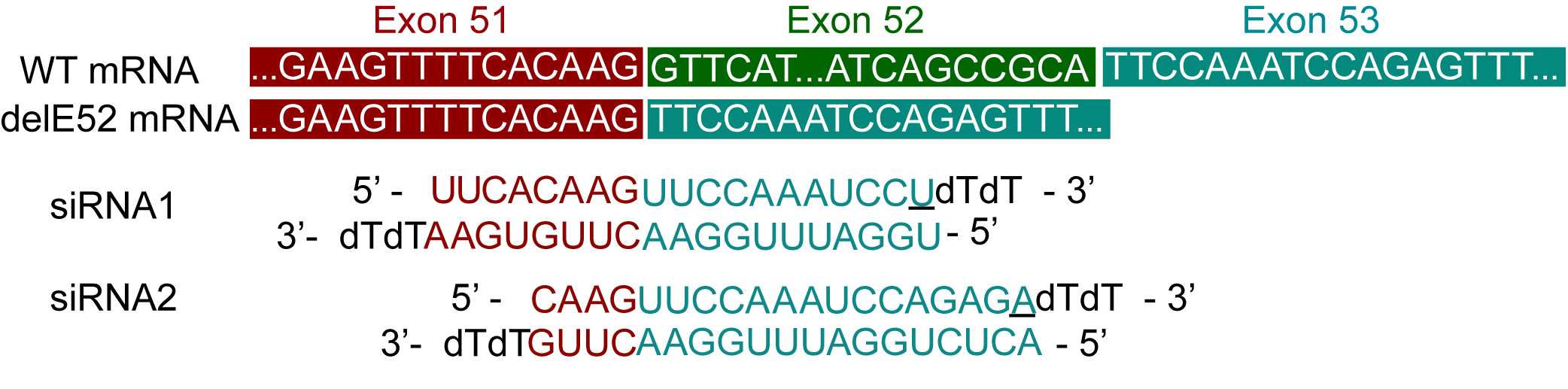

Supplement: Supplementary file 1 — Figure S1. Schematic of COL12A1 exons 51–53 and sequence of two siRNAs designed to specifically target exon 51‐53 junction, the mutant mRNA product in family 1. [file ACN3-6-1980-s001.tif]

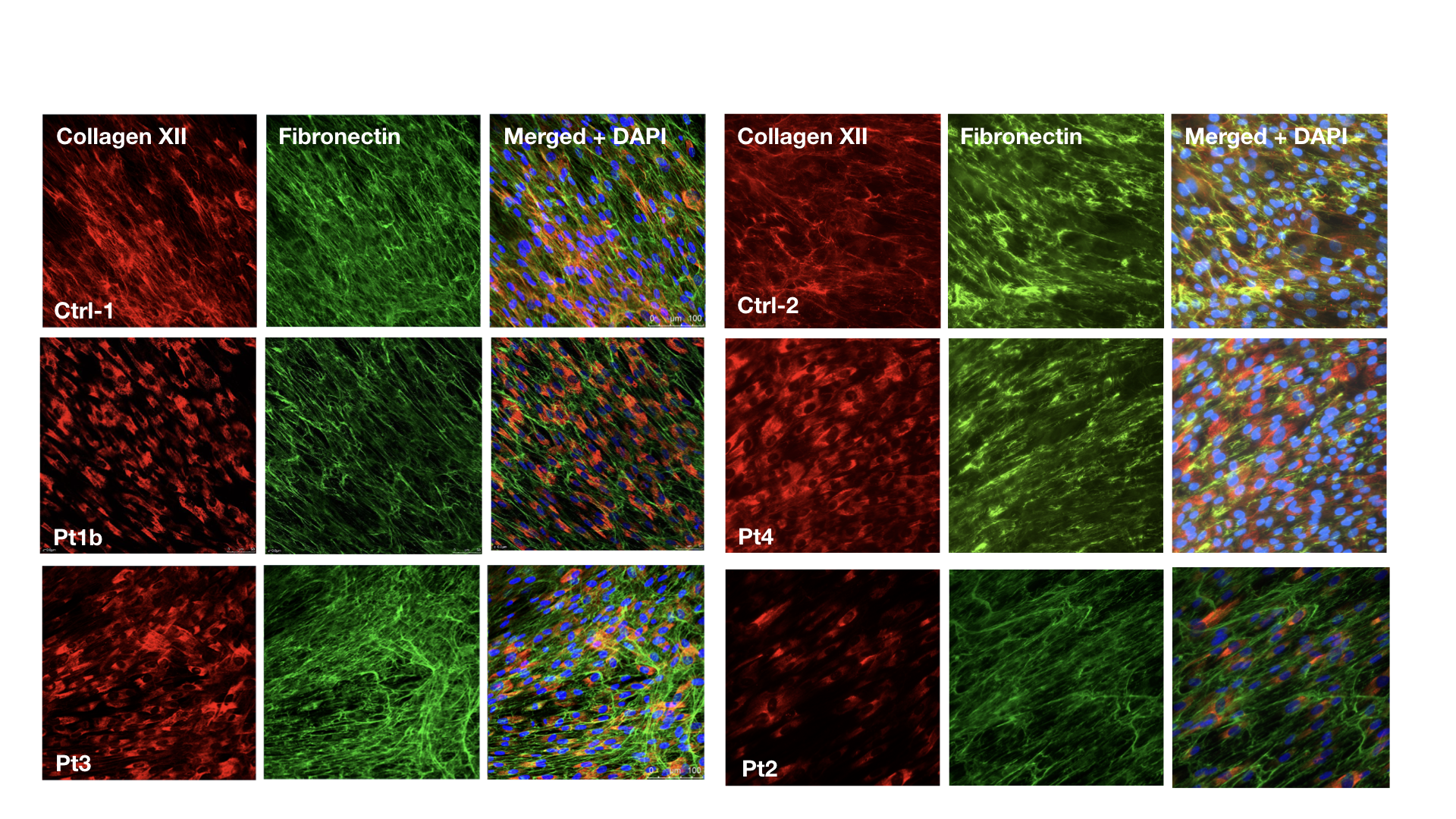

Supplement: Supplementary file 2 — Figure S2. Immunofluorescent images of skin dermal fibroblast cultures obtained from patient 1b, 2, 3, and 4 compared to two normal controls. There is a near complete absence of extracellular fibrillar collagen XII (red) staining with prominent intracellular retention of collagen XII when compared against other extracellular matrix proteins, for example, fibronectin (green). [file ACN3-6-1980-s002.tiff]
