# Supplementary material for: Dominant collagen XII mutations cause a distal myopathy
Source: Ann Clin Transl Neurol. 2019 Sep 11;6(10):1980–8. doi: 10.1002/acn3.50882 (PMC6801183; doi:10.1002/acn3.50882)
Supplement: Supplementary file 3 — Table S1. List of primers used for endpoint PCR and real‐time PCR (qPCR). [file ACN3-6-1980-s003.docx]

Supplementary Table 1. List of primers used for endpoint PCR and real-time PCR (qPCR)

| Primer | Sequence |
| --- | --- |
| gDNA-1F | 5’—ACTTTTATGTTTGAACTTTTCCAAG—3’ |
| gDNA-2F | 5’—GAAAGGCATTTGCATTCTTG—3’ |
| gDNA-3F | 5’—TTTGCTGCCAAACTTTTCAA—3’ |
| gDNA-4R | 5’—CCTGACCTCAAATGATCCAC—3’ |
| gDNA-5R | 5’—AGGGAGAAGTCCCAGAAACA—3’ |
| cDNA-E51F | 5’—CCAGAAACTCCCAGTGACCC—3’ |
| cDNA-E53R | 5’—CCACACTGGACTGCAGACAA—3’ |
| COL12A1-qPCR-F | 5’—AAAGGGGAAAGGAAATCAGC—3’ |
| COL12A1-qPCR-R | 5’—TCACAGCATCTGTCTCTACTGGT—3’ |
| COL12A1-del52-qPCR-F | 5’—TCACAAGTTCCAAATCCAGAGT—3’ |
| COL12A1-del52-qPCR-R | 5’—AGAGTCCATTGGGTCCCTGA—3’ |
| PGK1-qPCR-F | 5’—CAGCTGCTGGGTCTGTCAT—3’ |
| PGK1-qPCR-R | 5’—GCTGGCTCGGCTTTAACC—3’ |
