# Supplementary material for: Dominant collagen XII mutations cause a distal myopathy
Source: Ann Clin Transl Neurol. 2019 Sep 11;6(10):1980–8. doi: 10.1002/acn3.50882 (PMC6801183; doi:10.1002/acn3.50882)
Supplement: Supplementary file 4 — Table S2. Detailed description of clinical presentation, signs and symptoms, and relevant laboratory findings of patients with heterozygous, dominant, COL12A1 pathogenic variants. [file ACN3-6-1980-s004.docx]

Supplementary Table 2. Detailed description of clinical presentation, signs and symptoms, and relevant laboratory findings of patients with heterozygous, dominant, *COL12A1* pathogenic variants

­­

| **Family** | **1** | | | **2** | **3** | **4** |
| --- | --- | --- | --- | --- | --- | --- |
| **Patient** | 1A | 1B | 1C | 2 | 3 | 4 |
| **Mutation** | Heterozygous c.7951-630_8100+991del1771ins10 | | | Heterozygous c.8276G>A p.Gly2759Asp | Heterozygous c.8453G>A, p.Gly2818Glu | Heterozygous c.8065 G>A, p.Gly2689Arg |
| **Sex** | M | F | M | M | M | M |
| **Age at last exam** | 5 | 37 | 33 | 62 | 4 | 3 |
| **Age at Onset** | Congenital | Congenital | Congenital | 4 years old | Congenital | Congenital |
| **First symptom or sign** | Hypotonia, congenital hip dislocations, adducted thumbs | Hypotonia, hip dysplasia | Hypotonia, Hip dysplasia | Slow runner with subsequent improvement and became weak again at age 56 | Oligohydramnios, decreased fetal movements, hypotonia, abnormal foot positioning, torticollis and feeding difficulty in infancy | Congenital hypotonia, feeding difficulties, delayed motor milestones noted at 3 months of age |
| **Motor Development** | Gross motor developmental delay: rolled over by 6 months, walked by 2 years | Mild gross motor developmental dealy, head control was delayed, walked at 14 months of age; difficulty climbing stairs | Mild gross motor developmental delay. Could not ride a bicycle till 10 years of age | Normal | Gross and fine motor delay: pincer grasp at 1 years, finger fed at 2 years. Rolled over by 6 months, walked by 14 months with frequent falls. He can run and jump but does not hop, fatigues easily and wants to be carried. He does not alternate climbing up steps, needs assistance going down. | Gross and fine motor delay: Head control at 6 months, Rolled over by 10 months, sitting and crawling at 12 months, walked by 17 months; at 3yo, still has difficulty with stairs (does not alternate climbing up steps); kick a ball and jump at 3 years; expressive speech delay with limited words, some speech in sentences at 2 years |
| **Pattern of Weakness (MRC grade)** | Neck flexion 2, Deltoid 4, biceps 4, triceps 4, Hip flexion 4, knee flexion 4, knee extension 4, ADF 4 | Wrist flexion 4+, Finger extension, Finger spreading 4+, , Foot eversion 4+, toe flexion 4+ | Finger extension 4, ADF 4+, eversion 4+, toe flexion 4; difficulty walking on heels | Finger extension 4, finger flexion 4+, APB 4-, FDI 4, ADM 4+, ankle dorsiflexion 4+ (right)/4 (left), foot eversion 5 (right)/ 4 (left), toe extension 5 (right)/ 3 (left), toe flexion 5 (right)/ 4 (left); difficulty walking on heels | Does not comply with motor exam but has more than antigravity strength throughout. He is able to flex his neck against resistance, sit and stand from supine, squat, jump and run | Unable to obtain reliable MRC; approximately 4/5 throughout; mild head lag; torso weakness (must roll prone to rise from lying supine); uses 2 arms to stand from seated on ground; able to jump and run, with minimal ground clearance; reduced arms swin on run |
| **Contractures** | Ankles (Mild) | None | None | None | None | None |
| **Joint Hyperlaxity** | Small joints of the hand, knees, shoulders | Small joints of the hands, shoulders, knees | Wrists, hands | None | Distal and proximal. Small joints of the hand, wrists, elbows, knees, hips | Distal and proximal, noted in fingers (thumb) and toes; shoulders and fingers |
| **Other Clinical Findings** | Pes planus | Pes planus | Pes planus, post traumatic finger flexion contractures digit IV and V left hand | Pes cavus and calf atrophy | Expressive>receptive speech delay, non-verbal, velopharyngeal insufficiency. History of hypospadias and undescended testicles. Pes planus. Hyperkeratosis pilaris over extensor arm surface, normal scarring. | micrognathia, retrognathia; Expressive>receptive speech delay; minor pectus excavatum; lumbar lordosis with protuberant abdomen; flat feet (arch) with prominent posterior calcaneus; numerous small hyperpigmented birth marks on arms, legs and trunk; |
| **Muscle Ultrasound** | Normal | VL: moderate; RF: atrophy, moderate ; peroneus>TA; Paraspinal moderate. | VL: mild, granular/mixed; RF mild; TA: mild granular, Gastroc: mild mixed; Hamstring mild mixed pattern. | Not done | RF: mild, granular/streaky; VL: mild to moderate, mixed; Sart/gracilis: moderate, granular; TA, Gastrocnemii and soleus: mild, mixed; Hamstrings mild to moderate, granular; Deltoid, biceps and triceps mild, mixed; Lumbar paraspinal: morderate, granular. | Right Side: Generally all muscles with Grade 1-2 increaed granular (vs mixed) echogenicity; RF moderate granular; VL mild mixed; Hamstrings moderate granular; TA moderate granular; Gastroc mild to moderate mixed pattern; deltoid (streaky), biceps (mixed) and triceps (granual) all mild mixed pattern |
| **PFT (FVC)** | 95% predicted | 104% predicted | 96% predicted | 115% predicted | 81% predicted | Not done |
| **CK** | 174 U/L | 60 U/L | 101 U/L | 106 U/L | 103 U/L | 99 U/L |
| **EMG/NCS** | Not Done | Normal NCS, early recruitment of myopathic MUAPs in EHL, FDI, VL, Biceps | Normal NCS, mild neurogenic changes in APB, mild myopathic changes in FDS, EDC | Normal NCS, mild myopathic changes in EIP and thoracic paraspinals, and large motor unit potentials without reduced recruitment in other distal upper and lower limb muscles; brief runs of myotonic discharges in some distal muscles | Sedated at 3 years: normal sensory NCS. Motor NCSs showed slightly decreased CMAP amplitudes in the median and ulnar motor nerves. Normal peroneal and tibial motor NCSs. Increased insertional activity in the tibialis anterior, gastrocnemius, FDI and deltoid muscles. | Not done |
| **Echocardiogram** | Normal | Normal | Normal | Normal | Normal | Normal |

­
